# Silicon-nitride-based entrance slit design for the high-power-density monochromator in TPS 45A

**DOI:** 10.1107/S1600577523006240

**Published:** 2023-08-18

**Authors:** Ming-Ying Hsu, Huang-Wen Fu, Hok-Sum Fung, Chih-Yu Hua, Liang-Jen Huang, Huang-Ming Tsai

**Affiliations:** a National Synchrotron Radiation Research Center, 101 Hsin-Ann Road, Hsinchu Science Park, Hsinchu, Hsinchu 30076, Taiwan; Uppsala University, Sweden

**Keywords:** monochromator, entrance slit, silicon nitride, stability

## Abstract

Details of the design and operational status of the silicon-nitride-based entrance slit installed in the Taiwan Photon Source (TPS) 45A beamline are given. The slit is a diamond blade edge etched onto a copper slit part, which is in thermal contact with the silicon nitride base. A stable slit opening smaller than 4 µm is achieved in TPS 45A. The beam size at the slit has a full width at half-maximum of 3 µm in the vertical direction with a power of 20 W. Additionally, a hard stop made of invar is incorporated to control the thermal expansion displacement. The slit reduces the size and increases the stability of the source of the monochromator. Consequently, a higher energy resolution and excellent beamline stability are achieved.

## Abstract 2

Details of the design and operational status of the silicon-nitride-based entrance slit installed in the Taiwan Photon Source 45A beamline are given.

## 1. Introduction

The Taiwan Photon Source (TPS) 45A beamline is a sub-micrometre soft X-ray spectroscopy beamline collaboratively developed by the National Synchrotron Radiation Research Center (NSRRC) and the Max Planck Institute for Chemical Physics of Solids (MPI CPfS). The beamline enables a wide range of synchrotron-based spectroscopies to be performed, which are useful for uncovering the electronic structure of novel strongly correlated materials. Additionally, the TPS 45A is utilized to develop new synchrotron-based spectroscopic methods that are suitable for studying strongly correlated materials.

**Figure 1:**
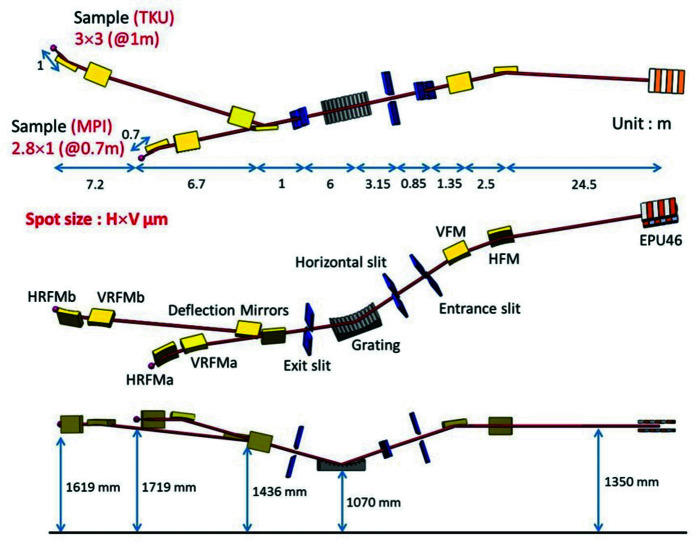
Optical design of the TPS 45A.

The TPS 45A beamline incorporates an elliptically polarized undulator with a 46 mm magnet period to generate photons with linear (horizontal and vertical) and circular (left and right) polarizations over an energy range of 280–1500 eV. The beamline’s optical design is based on the dragon-type design (Chen & Sette, 1989 ▸), with innovative features such as a bendable vertical focusing mirror (VFM) and an active grating monochromator (AGM) equipped with 25 actuator optical surface benders. The optical design of the TPS 45A beamline is illustrated in Fig. 1 ▸ (Tsai *et al.*, 2019 ▸). Its first mirror is the horizontal focusing mirror (HFM), which absorbs high-order energy to reduce the thermal load in subsequent optical components of the beamline. The VFM not only provides a high-degree polynomial profile to eliminate optical aberrations but also reduces intrinsic slope errors and thermal deformations on the optical surface. The VFM controls the vertical beam size at the AGM entrance and focuses the light onto the entrance slit. The AGM entrance slit is the third optical component and it receives the light that has passed through the HFM and VFM. Next, the light passes through the AGM entrance slit and the horizontal slit before entering the active grating mirror, which separates the different energy components of the light in the vertical direction. Subsequently, the light passes through the active grating mirror and is focused on the exit slit. The monochromated light then passes through additional mirrors, including horizontal refocus mirror a (HRFMa), horizontal refocus mirror b (HRFMb), vertical refocus mirror a (VRFMa) and vertical refocus mirror b (VRFMb), to focus the light at endstations a and b. The TPS 45A optical system ensures a high photon throughput of 2 × 10^11^ photons s^−1^ (0.01% bandwidth)^−1^ and an ultra-high-energy resolving power of 25000.

**Figure 2:**
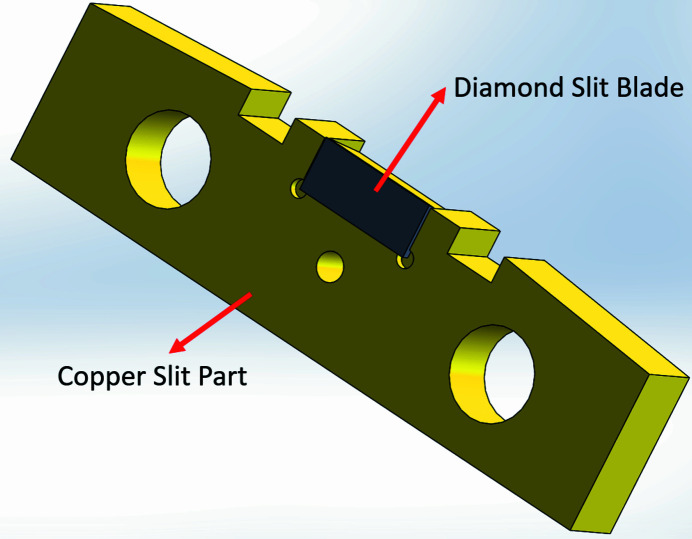
Diamond slit blade and copper slit part.

**Figure 3:**
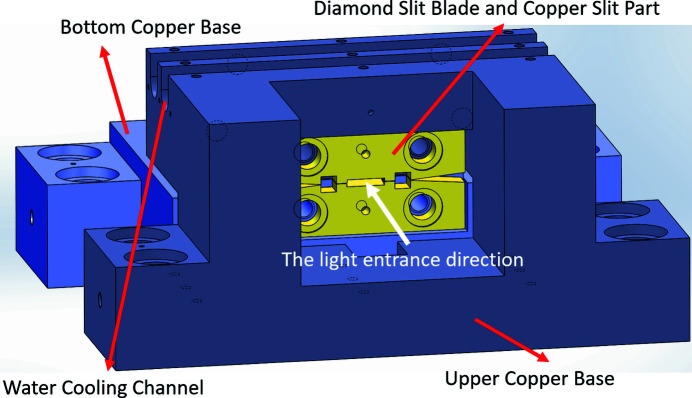
Original design for the diamond slit.

The AGM entrance slit is required to withstand high thermal loads while maintaining a small size in order to achieve high-energy-resolution output from the monochromator, which in turn increases the thermal load at the entrance slit. The entrance slit blade is made of diamond and has a thickness of 0.5 mm, a width of 7 mm and a height of 8 mm, as shown in Fig. 2 ▸. The original slit design comprises three parts: the upper copper base, the bottom copper base and two diamond slit blades integrated with a copper slit part, as shown in Fig. 3 ▸. Both the upper and bottom copper bases contain two water-cooling channels for accommodating the copper cooling tube. The water-cooling system maintains a stable slit opening by dissipating heat from the copper base. The diamond slit blades and the copper slit part must be insulated from the copper base in order to measure the photon current. Therefore, EP30AN-1 insulating glue is employed with a thickness of approximately 0.3 mm. The thermal conductivity of the EP30AN-1 glue is 3.31–3.61 W m^−1^ K^−1^, which is more than 100 times smaller than that of oxygen-free copper, 391 W m^−1^ K^−1^. The difference in thermal conductivity between the glue and copper results in a large temperature gradient at the glue layer.

The focus spot at the entrance slit has a full width at half-maximum (FWHM) beam size of ∼3 µm in the vertical direction and ∼50 µm in the horizontal direction. The total energy at the focus spot is 20 W. In the original entrance slit design, when the slit opening size is smaller than 7 µm, the opening size would vary over time. As a result, the AGM entrance slit opening size became unstable, leading to irregular energy resolution and photon flux output from the monochromator, thereby affecting the experimental results at the endstation.

This study aims to improve the thermal stability of the AGM entrance slit by redesigning the copper base. The contact area between the copper base and the diamond slit blade is replaced with a silicon nitride component, as silicon nitride is an insulating material and has a lower coefficient of thermal expansion (CTE) than copper. This modification resulted in a stable slit opening at the AGM entrance (Vašina *et al.*, 2001 ▸; Fabris *et al.*, 2019 ▸).

## 2. Simulation model

**Figure 4:**
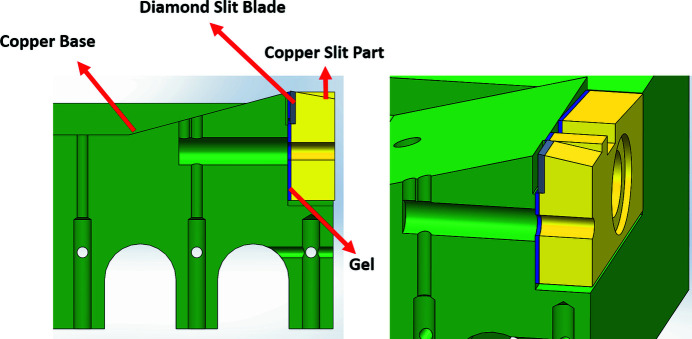
Cross-sectional and isometric views of the original design.

**Figure 5:**
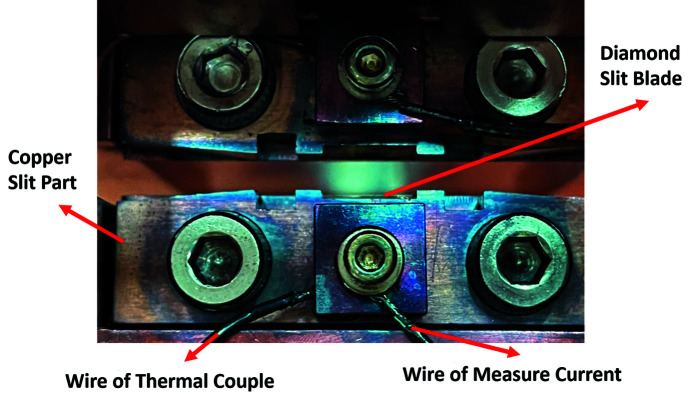
Original design of the diamond slit blade, the copper slit part, the thermal couple wire and the current measurement wire.

Figure 4 ▸ shows a cross-sectional view of the original design of the monochromator entrance slit system. The insulating glue was used to separate the diamond slit blade and the copper slit part from the copper base, while the diamond slit blade blocked a portion of the light to aid in detecting the photon current. The major components of the original design of the monochromator entrance slit system installed in the beamline, including the diamond slit blade, the copper slit part, the thermal couple wire and the current measurement wire, are shown in Fig. 5 ▸.

**Figure 6:**
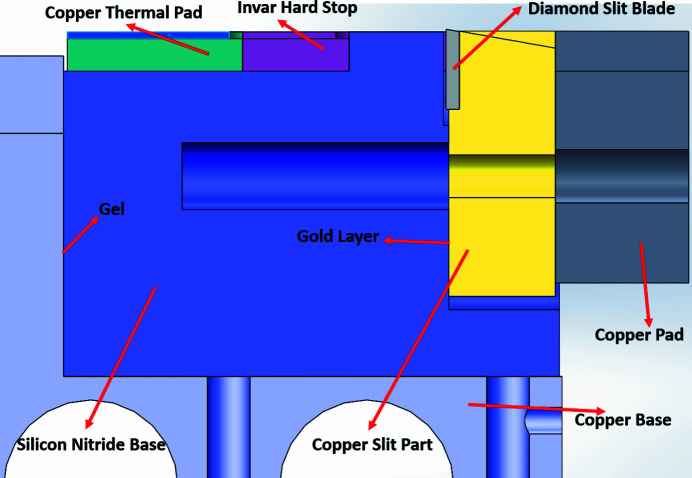
Cross-sectional view of the silicon nitride design.

Figure 6 ▸ shows a cross-sectional view of the silicon-nitride-base design. The silicon nitride base consisted of several additional parts, including the copper thermal pad, the invar hard stop, the gold layer and the copper pad, which increase its thermal stability and reduce its maximum temperature. Since the thermal expansion coefficient of silicon nitride is only 3.3 × 10^−6^ °C^−1^, roughly five times smaller than that of oxygen-free copper at 17.0 × 10^−6^ °C^−1^, it was not necessary to replace the entire copper base with silicon nitride, which would increase manufacturing complexity and cost. Instead, the silicon nitride base was designed as a modular component, allowing for the replacement of both the upper and bottom bases with the same silicon nitride base. After some minor modification of parts, the copper base used in the beamline was upgraded to a silicon nitride base.

In this study, some parts of the upper and bottom copper bases were replaced with a silicon nitride base in the high thermal concentration area. As an insulating material, the silicon nitride base was allowed to be in direct contact with the diamond slit blade and copper slit part.

**Figure 7:**
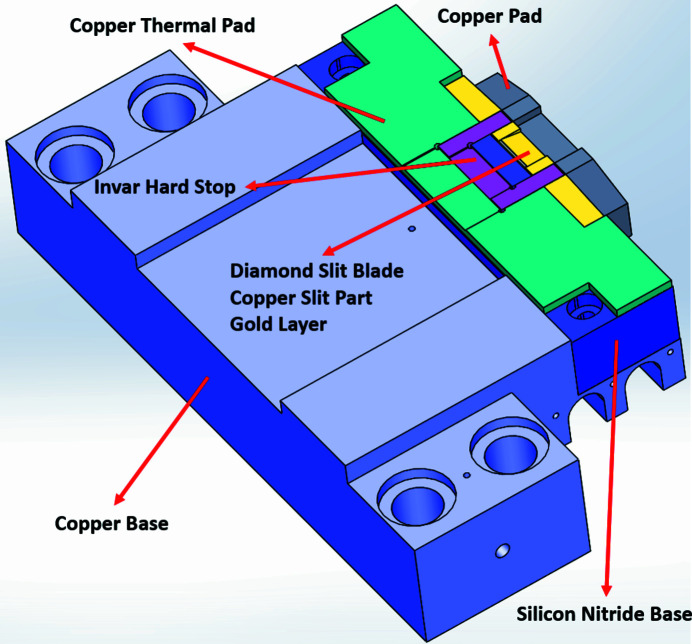
Top view of the silicon nitride base and additional parts.

**Figure 8:**
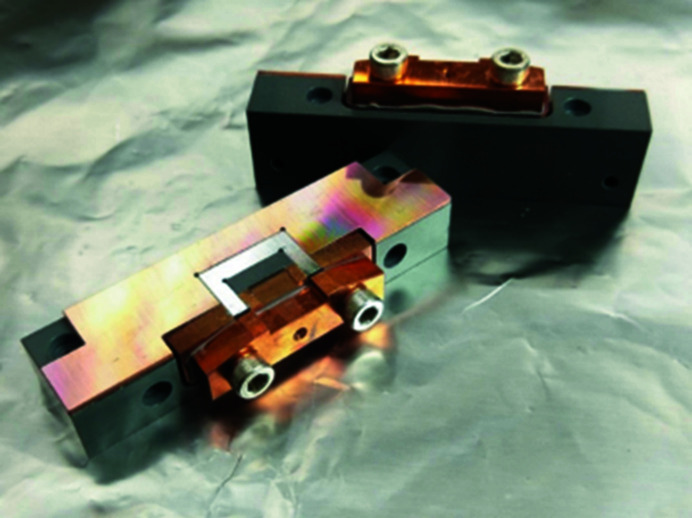
The silicon nitride base (made by Krosaki Harima) system with all accessory parts.

To improve the contact ratio between the two surfaces, EP30AN-1 glue was applied at the interface between the silicon nitride base and the copper base, which filled micro gaps and enhanced contact between the two parts. The thickness of EP30AN-1 between the two surfaces was less than 10 µm, ensuring low thermal conductivity without compromising thermal efficiency. Moreover, a gold layer was introduced on the contact surface to further enhance the contact ratio between the copper slit part and the silicon-nitride-base surface. Gold has a thermal conductivity of 310 W m^−1^ K^−1^ and is a soft metal, so it provides good contact between the two surfaces. To reduce the maximum temperature and thermal gradient of the diamond slit blade, a copper pad was placed in front of the copper slit part, which increased its thermal mass, stored heat and provided resistance to temperature fluctuations. The top of the silicon nitride base was designed with an invar hard stop to limit thermal expansion of the copper slit part during heat absorption by the diamond slit blade. The invar exhibits a thermal expansion of 1.6 × 10^−6^ °C^−1^ and a thermal conductivity of 12 W m^−1^ K^−1^. Furthermore, a copper thermal pad on the top of the silicon nitride base facilitates the distribution of thermal heat from the contact area with the diamond slit blade to the top surface of the silicon nitride base, which decreases the diamond slit blade’s maximum temperature and thermal gradient. Fig. 7 ▸ shows a top view of the silicon nitride base and additional parts, while Fig. 8 ▸ shows the silicon nitride base following assembly of all accessory components, including the invar hard stop, the copper thermal pad and the copper pad.

## 3. Thermal analysis

The silicon nitride base was installed in the modified copper base. The copper base was cooled by water flow in the copper pipe; the water flow rate was 2 L min^−1^, and the thermal convection coefficient was the critical parameter for the simulation. The forced convection factor for turbulent flow (Dittus & Boelter, 1930 ▸) inside the circular tube was calculated using several dimensionless numbers, namely the Reynolds number, the Prandtl number and the Nusselt number. The Reynolds number (Re) is defined as the ratio of fluid momentum force to viscous shear force. A lower Re indicates viscous forces are dominant and a higher Re indicates inertial forces are dominant. In general, laminar flow occurs when Re < 2300 and turbulent flow occurs when Re > 2900 (Holman, 2002 ▸). Re is calculated by

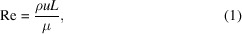

where ρ is the density of the fluid (kg m^−3^), *u* is the flow speed (m s^−1^), *L* is a characteristic length parameter in this study and denotes the internal pipe diameter (m), and μ is the dynamic viscosity of the fluid (N s m^−2^). The Prandtl number (Pr) is the ratio of momentum diffusivity (kinematic viscosity) to thermal diffusivity and is calculated as follows,

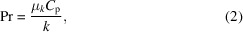

where μ_*k*_ is the kinematic viscosity of the fluid (× 10^−6^ m^2^ s^−1^), *C*_p_ is the heat capacity of the fluid (J kg^−1^ K^−1^) and *k* is the thermal conductivity of the fluid (W m^−1^ K^−1^). The Nusselt number (Nu) is the ratio of convective to conductive heat transfers across a boundary and is calculated as follows,

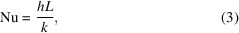

where *h* is the convection heat transfer (W m^−2^ K^−1^), *L* is the characteristic length parameter, and *k* is the thermal conductivity of the fluid (W m^−1^ K^−1^). After Re and Pr are calculated, they can be used in the Dittus–Boelter equation to solve for Nu. The Dittus–Boelter equation has two different conditions that need to be considered and these are calculated by

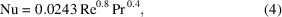

and

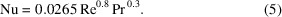

When the pipe wall temperature is higher than the fluid temperature, equation (4) is used, and, when the pipe wall temperature is lower than the fluid temperature, equation (5) is used. The Dittus–Boelter equations (4) or (5) can then be used in equation (3) to solve for the Nu number. The Nu number can then be used for the calculation of the convection heat transfer as follows,

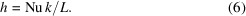

In the case of the monochromator entrance slit, the cooling water flow rate was 2 L min^−1^, the copper pipe diameter *L* was 4 mm, the water density ρ was 997 kg m^−3^ (at 20°C), the water viscosity μ was 0.001 Pa s (at 20°C), and the water flow velocity inside the pipe *u* was 2.65 m s^−1^. By substituting these values into equation (1), the Reynolds number can be calculated as Re = 10578.

At 20°C, the water’s kinematic viscosity was 1.0035 × 10^−6^ m^2^ s^−1^, its heat capacity was 4184 J kg^−1^ K^−1^ and its thermal conductivity *k* was 0.598 W m^−1^ K^−1^. The Prandtl number has no length scale factor and is solely determined by the fluid and its state. The Prandtl number was 7 and this value was used in equation (2).

After the Reynolds and Prandtl numbers are calculated using equations (1) and (2), they can be substituted into equation (4) to solve for the Nusselt number. The Dittus–Boelter equation is valid for smooth pipes. A suitable range for the Reynolds number is Re ≥ 10000 and the Prandtl number should be 160 ≥ Pr ≥ 0.6, with *L*_p_/*L* ≥ 10, where *L*_p_ is the copper pipe’s length and *L* is the copper pipe’s diameter. In this study, Re was 10578 and Pr was 7. The copper pipe was a hard-drawn copper pipe with a smooth wall surface, with *L*_p_/*L* ≃ 200. These conditions satisfy the requirements for applying the Dittus–Boelter equation. As a result, the Nusselt number is 87.74.

Equation (3) can be rewritten to equation (6) by substituting the values of Nu (87.74), thermal conductivity of water *k* (0.598 W m^−1^ K^−1^ at 20°C), and the diamoeter of the copper pipe *L* (4 mm). The convection heat transfer was 13116.74 W m^−2^ K^−1^. Convection heat transfer is essential for calculating heat absorption from the light source and provides an estimate for the maximum temperature of the monochromator entrance slit.

The simulation uses the finite-element method (FEM) to determine the thermal distribution of the monochromator entrance slit. The beam heat load applied at the edge of the diamond slit blade was 20 W and the cooling water temperature was 20°C.

## 4. Simulation and system installation results

The simulation was conducted to estimate the temperature distribution at the monochromator entrance slit resulting from the heat absorption caused by the diamond slit blade cutting the beam spot. The temperature distribution results from the simulation can be used as a boundary condition for the static structure analysis using the FEM. The heating power at the edge of the diamond slit was 20 W and the convection heat transfer coefficient was calculated based on the thermal analysis described earlier, resulting in a value of 13116.74 W m^−2^ K^−1^ at the contact area with the water-cooling channel.

**Figure 9:**
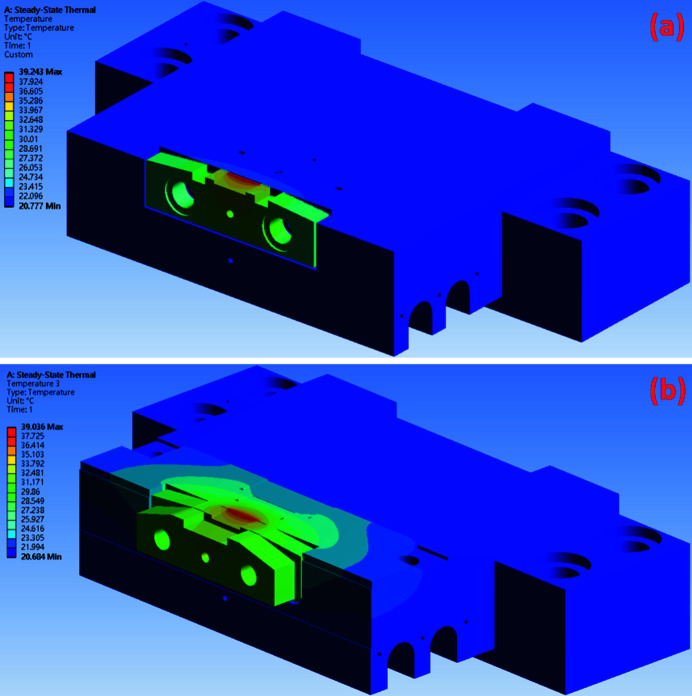
The temperature distribution of the monochromator entrance slit. (*a*) In the original design the maximum temperature is 39.24°C and (*b*) in the silicon-nitride-base design the maximum temperature is 39.04°C.

**Figure 10:**
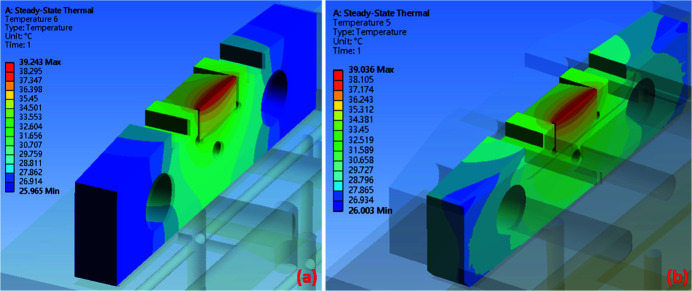
The temperature distribution of the diamond slit blade and copper slit part. (*a*) In the original design the maximum temperature is 39.24°C and (*b*) in the silicon-nitride-base design the maximum temperature is 39.04°C.

The temperature distributions for the original design and the silicon-nitride-base design are shown in Figs. 9 ▸(*a*) and 9(*b*), respectively, using the results of simulations conducted with *Ansys* software (https://www.ansys.com). In the original design, the maximum temperature is achieved in the region of the diamond slit blade, with a value of 39.24°C. In the silicon-nitride-base design, the maximum temperature is 39.04°C. These thermal simulation results show that the maximum temperature in the silicon-nitride-base design is slightly lower than that in the original design. However, the thermal conductivity of silicon nitride is lower than that of oxygen-free copper. The copper pad and diamond slit blade are considered high-temperature regions in the monochromator entrance slit. The simulation results for the original design and the silicon-nitride-base design and their maximum temperature zones in the edge of the diamond slit blade are shown in Figs. 10 ▸(*a*) and 10(*b*), respectively. The maximum temperature at the diamond slit blade in the original design is 39.24 °C, while that in the silicon-nitride-base design is 39.04°C. Thus, the silicon-nitride-base design exhibited a slightly reduced maximum temperature compared with the original design.

**Figure 11:**
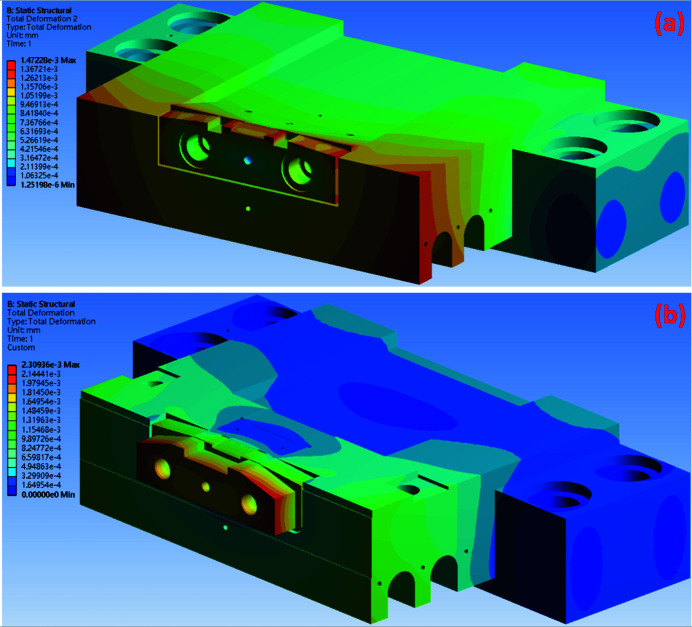
The total deformation of the monochromator entrance slit. (*a*) In the original design the maximum total deformation is 1.47 × 10^−3^ mm and (*b*) in the silicon-nitride-base design the maximum total deformation is 2.31 × 10^−3^ mm.

**Figure 12:**
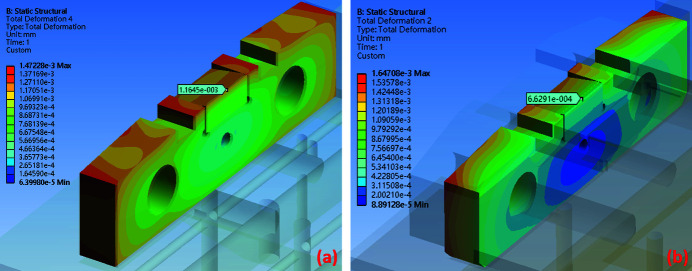
The total deformation of the copper slit part and diamond slit blade. (*a*) In the original design the maximum total deformation is 1.47 × 10^−3^ mm and the deformation in the region of the diamond blade edge is 1.16 × 10^−3^ mm and (*b*) in the silicon-nitride-base design the maximum total deformation is 1.65 × 10^−3^ mm and the deformation in the region of the diamond blade edge is 6.63 × 10^−4^ mm.

The thermal simulation results can be used as temperature boundary conditions for the static structural analysis with the FEM simulation conducted using *Ansys* software. The maximum total thermal deformation in the original design is 1.47 × 10^−3^ mm and that for the silicon-nitride-base design is 2.31 × 10^−3^ mm, as shown in Figs. 11 ▸(*a*) and 11(*b*). The maximum thermal deformation in the original design occurs in front of the copper base, the copper slit part and the diamond slit blade, whereas that in the silicon-nitride-base design occurs in the copper pad area. The thermal deformation in the copper slit part of the silicon-nitride-base design was not significant. Therefore, comparing the copper slit part and the diamond slit blade in both designs reveals that the maximum deformation in the original design case is smaller than that in the silicon-nitride-base design, with a difference of 0.49 × 10^−3^ mm, as shown in Figs. 12 ▸(*a*) and 12(*b*). However, the region of the edge of the diamond slit blade exhibits a maximum thermal deformation of 0.497 × 10^−3^ mm (Fig. 12 ▸), which is smaller in the silicon-nitride-base design compared with the other case.

**Figure 13:**
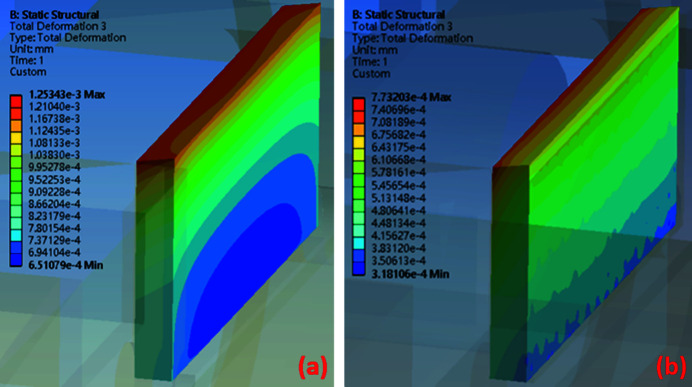
The total deformation of the diamond slit blade. (*a*) In the original design the maximum total deformation is 1.25 × 10^−3^ mm and (*b*) in the silicon-nitride-base design the maximum total deformation is 7.73 × 10^−4^ mm.

The deformation of the diamond slit blade is a crucial factor that affects the opening size of the monochromator entrance slit. In the original design the diamond slit blade exhibits a thermal deformation of 1.25 × 10^−3^ mm, while in the silicon-nitride-base design this deformation decreased to 7.73 × 10^−4^ mm, as shown in Figs. 13 ▸(*a*) and 13(*b*). The thermal deformation of the edge of the diamond slit blade in the silicon-nitride-base design significantly decreased by approximately 38% compared with the original design.

**Figure 14:**
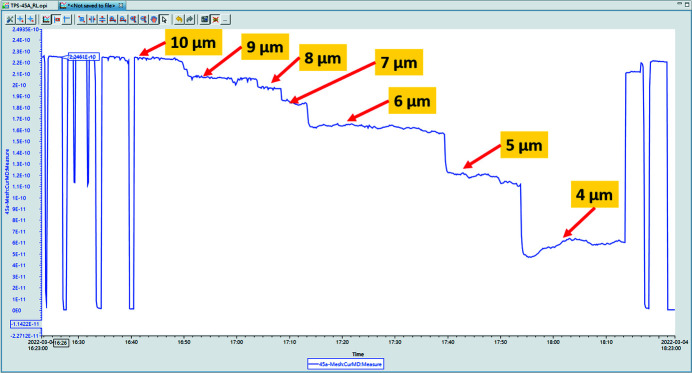
Photon current over time at the monochromator entrance slit.

After being installed in the TPS 45A, the monochromator entrance slit with the silicon nitride base underwent testing and the results are shown in Fig. 14 ▸. The *X*-axis represents time and the *Y*-axis represents the photon flux. The results show that the photon flux remained constant despite variations in the opening size of the monochromator entrance slit.

**Figure 15:**
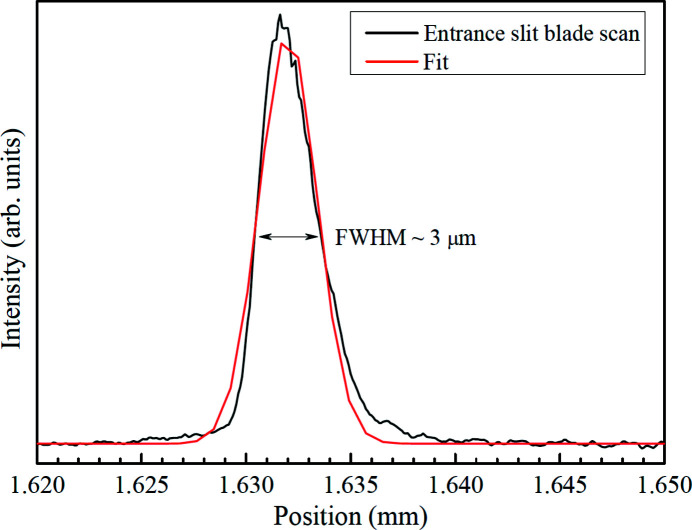
Focus spot size at the entrance slit.

As shown in Fig. 15 ▸, the FWHM of the focus spot size at the entrance slit was approximately 3 µm. The entrance slit controls the spot size of the monochromator entrance and the silicon nitride keeps the opening size of the entrance slit stable under thermal load. In the original design, the entrance slit gradually closed over time because of the thermal load.

## 5. Conclusions

The TPS 45A is a sub-micrometre soft X-ray spectroscopy beamline collaboratively developed by the NSRRC and MPI CPfS. The energy resolution is a crucial factor in determining the beamline’s performance, and the opening size of the entrance slit plays a critical role in achieving a high energy resolution. The monochromator entrance slit must be able to withstand the thermal load and maintain a constant opening size.

This study improved on the original copper-base monochromator entrance slit by using a silicon nitride base. The silicon nitride base ensured a constant opening size of the monochromator entrance slit across various energy resolutions. The results of this study can be applied to other similar soft X-ray spectroscopy beamlines at the TPS, including the TPS 27A, 33A, 35A, 39A and 43A, which also have a silicon-nitride-base design.
